# Acute liver failure caused by severe acute hepatitis B: a case series from a multi-center investigation

**DOI:** 10.1186/1476-0711-13-23

**Published:** 2014-06-23

**Authors:** Chun-ya Wang, Pan Zhao, Wei-wei Liu

**Affiliations:** 1 Emergency & Critical Care Center, Beijing Anzhen Hospital, Capital Medical University, Beijing 100029, China; 2 Clinical Trial Center, Liver Failure Therapy and Research Center, Beijing 302 Hospital (PLA 302 Hospital), No.100 of West Fourth Ring Middle Road, Beijing 100039, China; 3 Postgraduate Division, Academy of Military Medical Science, Beijing 100850, China

**Keywords:** Acute liver failure, Acute hepatitis B, Prognosis

## Abstract

**Background:**

Few data can be available regarding acute liver failure (ALF) caused by severe acute hepatitis B up to now. This study aims to report such cases from China.

**Findings:**

We conducted a multi-center investigation on ALF from 7 tertiary hospitals in different areas of China. A total of 11 patients with ALF caused by severe acute hepatitis B were finally identified. In these patients, there were 10 male and 1 female patients. As a serious complication, apparent hemorrhage occurred in 9 patients. Eventually, in these 11 patients, 4 survived and 7 died. 4 died of heavy bleeding, 2 died of systemic inflammatory response syndrome and 1 died of irreversible coma. No patients received liver transplantation.

**Conclusions:**

ALF caused by severe acute hepatitis B is worthy of formal studies based on its rarity and severity.

## Introduction

Hepatitis B virus (HBV) infection is a global health concern [1]. Acute HBV infection can cause severe acute hepatitis B that can rapidly progress to acute liver failure (ALF), which results in death or transplantation in 80% of relative individuals [2]. ALF is a life-threatening disease characterized by rapid deterioration of liver function in a patient without previously recognized liver disease. China is a particularly endemic area for HBV infection [3]; however, up to now, no extensive investigation on ALF has been carried out and thus no representative data are available in China. Recently, we performed a multi-center investigation of ALF in Chinese population and found out 11 cases with ALF caused by severe acute hepatitis B. Because of its rarity, studies on this disease are much fewer. Here, we reported the clinical features and prognosis of these cases.

### Patients and methods

#### Patient collection

**Figure 1:**
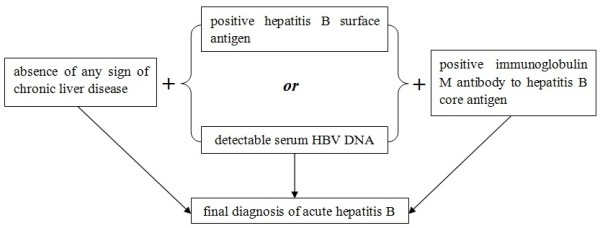
Diagnostic sketch of acute hepatitis B.

ALF in this study was defined as coagulopathy (prothrombin activity (PTA) ≤ 40% or international normalized ratio (INR) ≥ 1.5), jaundice (serum total bilirubin (TBil) ≥171 μmol/L) and encephalopathy (any degree of altered mentation) within 4 weeks in a patient without pre-existing liver diseases. The diagnosis of acute hepatitis B was based on the detection of HBsAg (or serum HBV DNA) and immunoglobulin M antibody to hepatitis B core antigen (Figure 1). Patients with ALF caused by severe acute hepatitis B between January 2007 and December 2012 were enrolled in this study. None of these patients had coinfection with hepatitis D virus.

Seven tertiary military hospitals in different areas of China were included in this investigation: Beijing 302 Hospital, PLA General Hospital, Changhai Hospital Affiliated to Second Military Medical University, PLA 161 Hospital, PLA 477 Hospital, General Hospital of Jinan Military Region and General Hospital of Lanzhou Military Region.

The study was performed in accordance with the ethical guidelines of the 1975 Declaration of Helsinki and was approved by the ethics committees of each hospital. Informed consent was obtained from all patients or guardians for being included in the study.

#### Data extraction and assessment

Demographic and clinical data were obtained from the electronic medical records and follow-up documents. Grade of HE had been determined in the medical records according to the widely-accepted following criteria in China [4]: I. loss of sleep rhythm, anxiousness, confusion or flapping tremors; II. loss of sphincter control, drowsiness or behavioral disorder; III. persistent coma, but still responding to shouts; IV. deep coma with no consciousness.

## Findings

### Demographic and clinical features on admission

Of the 11 patients, 10 were male and 1 was female, and 9 had positive and 2 had negative hepatitis B e antigen. None of these patients had the history of excessive drinking. Other demographic and entry laboratory characteristics of these patients including age, grade of HE, white blood cell count, hemoglobin, platelet count, PTA, INR, serum alanine aminotransferase (ALT), serum aspartate aminotransferase (AST), serum alkaline phosphatase (ALP), serum cholinesterase, serum lactate dehydrogenase (LDH), serum TBil, serum albumin, serum creatinine, serum urea nitrogen (UN), serum glucose, serum Na^+^, serum K^+^, serum Cl^−^ and arterial blood ammonia (BLA) are summarized in Table 1.

**Table 1:** Clinical characteristics of patients with ALF on admission and outcomes

**Parameters**	**Patient 1**	**Patient 2**	**Patient 3**	**Patient 4**	**Patient 5**	**Patient 6**	**Patient 7**	**Patient 8**	**Patient 9**	**Patient 10**	**Patient 11**
Sex	male	male	male	male	male	female	male	male	male	male	male
Outcome	death	survive	death	death	survive	survive	death	survive	death	death	death
Age (years)	70	27	28	58	50	55	44	26	39	47	47
Body Mass Index (kg/m^2^)	26.12	23.94	24.49	23.25	27.44	22.15	24.34	26.23	22.02	24.62	27.18
Grade of HE	4	2	3	3	1	3	3	1	3	4	3
Days from onset of illness to outcome (death or recovery)	16	10	4	3	14	20	10	11	7	4	10
Serum ALT (U/L)	1303	2000	4679	2917	941	1333	1645	3562	1164	1886	8294
Serum AST (U/L)	664	1700	5111	3975	758	447	922	2277	516	155	6486
Serum TBil (μmol/L)	225.2	463.1	173.5	180.6	386.3	173.6	216.0	179.0	194.2	173.9	215.0
Serum ALP (U/L)	183	144	129	217	175	159	222	164	202	144	266
Serum LDH (U/L)	230	138	1015	334	324	391	233	354	212	330	608
Serum Albumin (g/L)	33	32	31	35	28	25	28	40	37	29	38
Serum glucose (mmol/L)	7.6	2.5	12.1	17.5	2.3	3.8	11.0	4.8	3.1	10.5	4.3
Serum creatinine (μmol/L)	117	58	97	168	118	69	95	68	77	90	92
Serum UN (mmol/L)	4.8	3.3	3.0	1.9	6.1	2.5	1.8	1.5	4.3	1.7	2.7
Serum Na^+^ (mmol/L)	135	138	137	133	135	139	133	140	137	137	132
Serum K^+^ (mmol/L)	4.5	3.8	3.8	4.8	4.5	3.0	3.9	3.8	3.8	4.2	4.0
Serum Cl^−^ (mmol/L)	103.1	105.8	103.2	103.9	98.6	106.5	106.0	102.7	101.3	108.6	95.7
Serum HBV DNA (IU/mL)	5.02 × 10^4^	1.00 × 10^3^	6.32 × 10^8^	3.23 × 10^4^	2.63 × 10^5^	1.27 × 10^4^	3.22 × 10^7^	2.19 × 10^5^	6.20 × 10^9^	1.49 × 10^3^	1.54 × 10^3^
White blood cell count (×10^9^)	4.06	3.24	9.62	12.16	7.3	5.31	6.11	6.7	11.89	12.47	11.17
Platelet count (×10^9^)	149	122	87	88	97	44	66	147	120	29	38
Hemoglobin	129	103	138	144	137	105	127	151	138	140	148
PTA (%)	34	30	11	6.68	21.4	21.1	20	33	19.8	21	15
INR	1.60	1.92	3.53	5.49	2.52	2.39	2.77	1.63	2.40	2.45	2.89
Arterial BLA (μmol/L)	84	71	102	217	52	69	118	67	64	173	273

*ALF*, Acute liver failure; *HE*, Hepatic encephalopathy; *ALT,* Alanine aminotransferase; *AST*, Aspartate aminotransferase; total bilirubin; *ALP*, Alkaline phosphatase; *LDH*, Lactate dehydrogenase; *CHE*, Cholinesterase; *UN*, Urea nitrogen; *PTA*, Prothrombin activity; *INR*, International normalized ratio; *BLA*, Blood ammonia.

### Treatment, complications and outcomes

Patient 1, Patient 5 and Patient 10 received antiviral therapy with lamivudine and Patient 6 received antiviral treatment with entecavir at the diagnosis of acute hepatitis B. The other patients did not receive any antiviral treatment in the period of intensive care. Sera from Patient 5 and Patient 6 were sent to undergo the HBV reverse-transcriptase gene amplification and sequencing at the diagnosis with the method previously described by us [5], and no nucleos(t)ide-resistant mutant was reported.

During the clinical course, most significantly, serum TBil levels in non-survivors worsened during days 2 to 6 after admission, while levels in survivors improved during the same period. Other indicators did not exhibit especially interesting features.

As serious complications, sepsis occurred in 3 patients and apparent hemorrhage could be observed in 9 patients, 3 (Patient 3, Patient 4 and Patient 10) of whom developed diffuse alveolar hemorrhage. In these 11 patients, 4 survived (Patient 2, Patient 5, Patient 6 and Patient 8) and 7 finally died. 4 died of hemorrhage, 2 died of systemic inflammatory response syndrome and 1 died of irreversible coma. No patients received liver transplantation.

## Discussion

Severe acute hepatitis B is an important cause of ALF worldwide [6]. In real-world practice, a tough but vital problem that physicians often encounter is the differentiation between acute hepatitis B and acute exacerbation of chronic HBV infection. In order to ensure the accuracy of diagnosis in this study, we looked through the past status of hepatitis B surface antigen for each included patient and excluded any patient who could be identified or seemed to have acute exacerbation of chronic HBV infection.

Overall, the morbidity of ALF was more common in the female than that in the male [7]. However, according to our study, men were affected by HBV-related ALF more often than women. Regarding the treatment, contradictions still exist among different studies [8]. A randomized controlled trial from India concluded that though lamivudine (100 mg per day) caused a greater decrease in levels of HBV DNA, it did not cause significantly greater biochemical and clinical improvement as compared to placebo in patients with severe acute hepatitis B [9]. Another study from China reported that early treatment with lamivudine led to a greater decrease in HBV DNA level, better clinical improvement and mortality improvement in patients with severe acute hepatitis B [10]. For our study, in the 4 patients who received antiviral therapy, 2 died and 2 survived, while in the other 7 patients who did not receive antiviral treatment, 5 died and 2 survived. Whether antiviral treatment is necessary for ALF caused by severe acute hepatitis B needs further extensive studies, but the obstacle is big because a randomized trial in the setting of severe acute hepatitis B seems unethical.

In this study, the mortality was very high and all patients who died had HE grade 3 to 4, suggesting a more advanced disease progression in these patients. Additionally, heavy bleeding is always the most frequent but refractory complication in patients with ALF [11]. Hemorrhage attributed to the main cause of mortality in this investigation. The only treatment option for hemorrhage is blood transfusion in China. However, shortage of blood usually leads to dismal outcomes. On the other hand, the rate of liver transplantation in this investigation was null, though it remained the effective treatment in ALF when standard medical therapy failed. The reasons for this included the difficulties in obtaining organs in urgent fashion, as well as the economic situation of patients in China. Shortage of transplantation also resulted in the high mortality of ALF.

In summary, we reported the rare clinical entity of ALF caused by severe acute hepatitis B. Hemorrhage was the most common complication and cause of death. For these critically ill patients, coagulopathy is a vital problem in the intensive care.

## Availability of data

The data supporting the results of this study are included within this article.

## Competing interests

The authors declare that they have no competing interests.

## Authors’ contributions

PZ designed the study; CW and PZ were involved in the data collection; WL checked the data; CW and PZ wrote the manuscript. All authors read and approved the final manuscript.
